# Clinical and multi-omics characterisation of early neurodevelopmental disorders associated with critical congenital heart disease: the prospective cohort CATAMARAN neonatal study protocol

**DOI:** 10.1136/bmjopen-2026-116866

**Published:** 2026-09-08

**Authors:** Oscar Werner, Véronique Ferchaud-Roucher, Matilde Karakachoff, Pierre Bourgoin, Anne Chauvire-Drouard, Jules Galy, Mathilde Cosse, Mélissa Chaffiraud, Marion Boivin, Evelyne Gauvard, Solène Egron, Bénédicte Romefort, Nadir Benbrik, Paul Padovani, Solene Prigent, Maha Tagorti, Marie Demonceaux, Céline Grunenwald Gronier, Jihed Laribi, Astrid Monier, Norbert Winer, Bénédict Gaillard Le Roux, Cyril Flamant, Valérie Amarger, Thomas Moyon, Hervé Blottière, Virginie Forest, Pierre Lindenbaum, Christian Dina, Richard Redon, Julien Barc, Frédéric Ebstein, Jean-Jacques Schott, Amanda Guerra, Olivier Cadeau, Arnaud Roy, Alban-Elouen Baruteau, CATAMARAN Study Group, Damien Bonnet

**Affiliations:** 1 CHU Nantes, Department of Pediatric Cardiology and Pediatric Cardiac Surgery, FHU PRECICARE, Nantes Université, F-44000 Nantes, France; 2 CHU Nantes, INSERM, Department of Pediatric Cardiology and Pediatric Cardiac Surgery, CIC FEA 1413, Nantes Université, F-44000 Nantes, France; 3 INRAE, UMR 1280, PhAN, Nantes Université, F-44000 Nantes, France; 4 CHU Nantes, Pôle Hospitalo-Universitaire 11: Santé Publique, Clinique des données, INSERM, CIC 1413, Nantes Université, F-44000 Nantes, France; 5 CHU Nantes, Department of Neonatal and Pediatric Intensive Care, Nantes Université, Nantes, France; 6 CHU Nantes, Department of Gynecology & Obstetrics, Nantes Université, Nantes, France; 7 CHU Nantes, CNRS, INSERM, l’institut du thorax, Nantes Université, F-44000 Nantes, France; 8 CHU Nantes, Centre Référent des Troubles d’Apprentissage, Nantes Universite, Nantes, France; 9 LPPL, SFR Confluences, Université d’Angers, F-49000 Angers, France

**Keywords:** Developmental neurology & neurodisability, Paediatric intensive & critical care, Prenatal diagnosis, GENETICS, Congenital heart disease

## Abstract

**Introduction:**

Critical congenital heart disease (CHD) is associated with neurodevelopmental disorders, recognised as the most common long-term morbidity in affected children. In critical CHD, that is, CHD requiring cardiac surgery within the first 3 months of life, 30%–50% of children have lower developmental scores. Therefore, early identification of at-risk infants is crucial, yet there is no scientifically evaluated care programme in France. This study aims to evaluate early neurodevelopmental status in infants with prenatally diagnosed critical CHD and to determine how intrinsic susceptibility, prenatal and postnatal factors are functionally associated with developmental delay in this population.

**Methods and analysis:**

Caractérisation et Accompagnement des Troubles du neurodéveloppement Associés aux MAlfoRmations cArdiaques coNgénitales (CATAMARAN) is a prospective, multicentre cohort study including 150 fetuses with critical CHD and their parents across eight French tertiary CHD centres. The primary objective will be to estimate the proportion of developmental delay at 6 months using the Bayley Scales of Infant and Toddler Development. Secondary objectives will include exploring potential prenatal, perinatal, perioperative determinants of developmental delay. Data collection will span pregnancy to 6 months of age including clinical assessments, maternal questionnaires (stress and nutrition), multimodal imaging and extensive biobanking (placenta, cord and peripheral blood, stool samples). To explore potential genetic and other multi-omic factors involved in the occurrence of a developmental delay, a case-control analysis will be conducted within the cohort.

**Ethics and dissemination:**

Clinical and biological data will be collected through a secure system, with anonymised samples analysed in specialised facilities under collaborative agreements. Data confidentiality, traceability and long-term storage are ensured through controlled access and audit trails. Study results will be published and shared with families and the public through the patient association Petit Coeur de Beurre. This study received approval from a French ethics committee in November 2024 (no. 2024-A00425-42).

**Trial registration number:**

NCT06690151.

**STRENGTHS AND LIMITATIONS OF THIS STUDY**

- The CATAMARAN study uses a prospective, multicentre, cohort design with embedded case-control multi-omics analyses, enrolling 150 family trios across eight French tertiary congenital heart disease (CHD) centres.
- The primary neurodevelopmental outcome is assessed using the Bayley Scales of Infant and Toddler Development, Fourth Edition, a norm-referenced, standardised, age-corrected tool validated in CHD and preterm populations.
- The biobanking protocol integrates six complementary biological matrices (maternal blood, placental tissue, umbilical cord blood, neonatal blood, paternal blood and stool) collected under standardised, accredited conditions (NF S96-900, ISO 9001).
- Because neurodevelopmental assessments at 6 months have inherent sensitivity limitations, participants will be followed longitudinally up to 24 months of age.
- The single-country (French) design and exploratory nature of multi-omics analyses limit the immediate generalisability of findings and will require replication in independent international cohorts.

## Introduction

 Congenital heart disease (CHD) is a critical healthcare issue, comprising a group of rare diseases (prevalence <1/2000 live births) that, collectively, are the primary cause of congenital defects, with a birth prevalence of 1%.^1^ Whereas only a minority of patients survived 50 years ago, currently >90% of patients reach adulthood following advances in prenatal diagnosis, surgical techniques and perioperative care.^2^ Therefore, CHD research needs to shift from improving mortality to decreasing morbidity. Neurodevelopmental disorders (NDD) have been recently recognised as the most common morbidity and long-term sequelae in patients with CHD across the lifespan.^3^ The prevalence and severity of NDD increase with the complexity of CHD. In critical CHD, that is, CHD requiring cardiac surgery within the first 3 months of life, 30%–50% of children have lower scores for cognition, language, attention, executive functions, as compared with their peers, with a long-lasting impact on academic achievement, transition to independence and quality of life.^4 5^ Early identification of at-risk infants is primordial as they would benefit the most from early preventive measures. However, while both the American Heart Association and the Association for European Paediatric and Congenital Cardiology have published clinical guidelines regarding the neurodevelopmental risk assessment in grown-up children which operated CHD,^4 6 7^ there is no funded, coordinated, durable and scientifically evaluated multicentric care programme focusing on CHD-associated NDD in France.^8^

Moreover, causes of CHD-associated NDD are mostly unexplained. Early assumptions that NDD were only due to perinatal neurological compromise or perioperative brain injury are now proved to be incorrect. Indeed, despite being multiple, synergistic and cumulative, postnatal risk factors account for <30% of cases, leaving NDD underexplained in over 70%—suggesting that CHD-associated NDD may be preprogrammed.^4 7^ This major breakthrough suggests that as-yet unidentified intrinsic and/or prenatal factors could play a key role. It opens new perspectives in translational research to solve those clinical issues. Among the various causes, genetic factors are a potential major contributor to brain dysmaturation in CHD and shared genetic pathways may alter both heart and brain development.^6 9 10^ Placental abnormalities may also impact brain development, and CHD-associated NDD could partly arise from a triangular interplay between the placenta, the fetal heart and the brain. It is consistent with the Developmental Origins of Health and Disease hypothesis, that is, the in utero programming of early and later-in-life non-communicable cardiometabolic and mental diseases.^7^

Therefore, the *CATAMARAN neonatal* study, which is a prospective cohort study with a case-control multi-omics analysis, aims to comprehensively assess the neurodevelopmental risk at 6 months of age in infants with critical CHD, and to determine how intrinsic susceptibility (ie, genomic variations), prenatal (ie, maternal fetal environment and placental dysregulation) and postnatal (ie, adaptation to extrauterine life and perioperative events) factors are functionally associated with developmental delay in this population.

## Methods

### Study objectives

The primary objective of the *CATAMARAN neonatal* study is to assess the prevalence of developmental delay at 6 months of age in infants with prenatally diagnosed critical CHD.

The secondary objectives are to identify intrinsic, prenatal, perinatal and perioperative determinants of CHD-associated developmental delay.

### General study design and settings

The *CATAMARAN neonatal* study is a multicentre, prospective cohort study involving critical CHD neonates and their two parents with a follow-up duration of 6 months and an expected recruitment time period of 24 months. Recruitment commenced in January 2026, with a planned end of recruitment in December 2027. The primary neurodevelopmental assessments (Bayley Scales of Infant and Toddler Development, Fourth Edition (BSID-IV) at 6 months) will therefore be completed by June 2028.

This is a clinical research study including a cohort and a biocollection, with minimal risk and constraint. It will be conducted in eight French tertiary surgical CHD centres: (1) CHU de Nantes (coordinating centre); (2) Hôpital Necker-Enfants Malades, Paris; (3) CHU de Bordeaux; (4) CHU de Marseille; (5) CHU de Toulouse; (6) CHU de Tours; (7) Hôpital Marie Lannelongue, Le Plessis-Robinson; (8) Hôpital Antoine Béclère, Clamart.

Data will be collected by a co-investigator and a clinical research coordinator at each centre. Patient data will be anonymised and assigned a unique subject number before being transmitted to the principal investigator and clinical research coordinator.

### Study population: patients with critical CHD

The *CATAMARAN neonatal* project will include 150 foetuses with a prenatal diagnosis of sporadic critical CHD and their two blood-related parents when applicable. Prior to any study procedure, written informed consent will be obtained from both parents, for themselves and on behalf of their expected child, following provision of clear and accurate verbal and written information by the attending physician. Consent forms comply with the requirements of the approving ethics committee (Comité de Protection des Personnes, no. 2024-A00425-42).

A CHD will be defined and classified according to the 2021 International Paediatric and Congenital Cardiac Code and the 11th revision of the International Classification of Diseases.^11^ Neonatal CHD surgery risk stratification will be determined by the Risk Stratification for Congenital Heart Surgery (RACHS-2) score.^12^ Cardiac anatomy will be determined based on fetal echocardiogram. The critical character of the CHD will be discussed and validated before inclusion during a team discussion involving a fetal cardiologist, a cardio-paediatrician and a cardiac surgeon.

CHD fetuses will not be included if they exhibit any of the following conditions: suspicion metabolic, genetic or syndromic disease known to be associated with NDD; suspected congenital infection; assisted reproductive technology with third-party reproduction (ie, donor gamete); parents considering pregnancy termination. No restriction on gestational age at delivery is applied: both term (≥37 weeks) and preterm neonates are eligible. Preterm delivery complicates approximately 15%–20% of critical CHD pregnancies and its exclusion would compromise external validity. Gestational age at delivery will be recorded prospectively and included as a covariate in all multivariate models; BSID-IV scores will be interpreted using age-corrected norms for infants born before 37 weeks. A sensitivity analysis restricted to term-born neonates is preplanned.

### Outcome measures

#### Primary outcome

The primary outcome is the proportion of infants with developmental delay at 6 months of age using the BSID-IV.^13^ The ‘Bayley Scales’ is a norm-referenced standardised tool, translated in French and that has been used in clinical practice and in large cohorts to assess developmental functioning in infants and young children aged 16 days to 42 months.^14 15^ It evaluates three key domains: cognitive (sensorimotor development, exploration and manipulation of objects, concept formation, memory), language (receptive and expressive communication) and motricity (fine and gross motricity). Each domain yields standardised scores (mean 100, SD 15), enabling comparison with normative data.

This comprehensive assessment of the development will be performed by a qualified child neuropsychologist using BSID-IV, blinded to the diagnoses, neuroimaging and clinical status of the child. A significant developmental delay will be diagnosed when a child scores at least 1.5 SD below the normative mean in one or more assessed domains.

#### Secondary outcomes

Outcomes related to clinical endpoints:

- Define the prevalence of developmental delay at 6 months in infants with critical CHD, according to the severity of CHD (type and surgical risk score).
- Describe altered developmental domains (cognitive, language and motricity).^15^
- Assess the impact of CHD-associated developmental delay on parental stress at key moments (perioperatively, and at 6-month follow-up) using the impact of event scale-revised (IES-R). The IES-R is a 22-item self-report questionnaire, translated in French population, with each item rated on a Likert scale from 0 (not at all) to 4 (extremely), yielding a total score ranging from 0 to 88. A total score >33 is commonly used as a threshold indicating probable post-traumatic stress disorder.^16^
- Assess the association between sociodemographic factors (eg, parental level of education, family history of NDD) and the presence of developmental delay.

Outcomes related to intrinsic susceptibility to NDD are as follows:

Genomic analyses will be conducted using two complementary, sequentially planned strategies with distinct scientific objectives:

- Phase I—PRIMARY genetic objective, within-study: rare de novo variant identification by whole-genome sequencing (WGS) of the affected infant and both unaffected biological parents (family-trio design—the methodological gold standard for rare variant discovery in sporadic congenital disease). The trio approach enables unambiguous identification of de novo variants without requiring large case-control sample sizes, and is adequately powered within the current 150-trio dataset to detect de novo variants in genes with prior biological plausibility.^6 10^ CATAMARAN will generate one of the largest CHD-NDD trio-WGS datasets to date in France.
- Phase II—SECONDARY genetic objective, federated network design—NOT a within-study power claim: common variant discovery (genome-wide association study; polygenic score analyses) requires sample sizes of several thousand, far beyond any single critical CHD cohort. The CATAMARAN biocollection is designed as a founding node of a federated national and international genomics network: once recruitment is complete, data will be pooled with other CHD-NDD biobanks in the collaborative network and from the European Reference Network for Rare and Low Prevalence Complex Diseases of the Heart (ERN GUARD-HEART), consistent with the strategy of the Paediatric Cardiac Genomics Consortium. This is a long-term secondary objective, independent of the primary and multi-omics aims of the current study, and no within-study power claim is made.

Outcomes related to acquired predisposing or protective factors for the neurodevelopmental risk are as follows:

- Assess the in utero exposure to maternal, obstetrical and fetal factors, and to look for associations with developmental delay at 6 months. It will include maternal psychological impact with the IES-R questionnaire that will be assessed prenatally^16^; maternal dietary habits that will be assessed using a food frequency questionnaire (FFQ) developed for the Étude Longitudinale Française depuis l’Enfance (ELFE) cohort.^17^ This semi-quantitative questionnaire evaluates the frequency of consumption of major food groups over a typical week and has been validated against 24-hour dietary recalls. It is designed to capture habitual dietary patterns in young children within the French population.
- Look for placental morpho-functional characteristics associated with CHD-associated developmental delay.
- Identify placental transcriptomic, placental and umbilical cord blood epigenetic signatures of developmental delay associated with critical CHD. Placental transcriptomic profiling (RNA-sequencing) will identify differentially expressed genes and dysregulated pathways—notably mitochondrial bioenergetics, inflammatory signalling and angiogenesis—comparing cases (developmental delay) versus controls (normal development), building on our group’s pilot data demonstrating placental mitochondrial dysfunction in critical CHD. Epigenomic profiling (whole-genome bisulphite sequencing for DNA methylation) will target differentially methylated regions at imprinted and developmentally regulated loci in placental biopsies and umbilical cord blood mononuclear cells—encoding potential in utero neurodevelopmental programming. To our knowledge, this constitutes the first systematic, prospective, multi-layer omics analysis of human placenta in critical CHD using neurodevelopmental outcome as the case-control stratifying variable.
- Characterise the maternal, fetal and placental metabolomic and lipidomic signatures of developmental delay associated with critical CHD. The lipidomic strategy is structured around a maternal-placental-fetal triadic design: (i) maternal peripheral blood (28–35 weeks of gestation), (ii) placental tissue (snap-frozen at delivery) and (iii) umbilical cord blood and early neonatal blood. This simultaneous, integrated profiling of three biological compartments, stratified by neurodevelopmental outcome in a prospective case-control design, is entirely novel: to our knowledge, no published study has characterised the full maternal-placental-fetal lipidomic continuum in critical CHD with NDD as the stratifying variable. Long-chain polyunsaturated fatty acids (LC-PUFAs), lysophospholipids and sphingolipids are critical mediators of trophoblast function, fetoplacental nutrient transfer, myelination and synaptic plasticity.^18 19^ Maternal dietary lipid intake (FFQ-ELFE), collected at the same time point as maternal blood sampling, will enable modelling of the dietary contribution to the lipidomic triad. All lipidomic analyses will be performed on the M-Shark mass spectrometry platform (INRAE UMR 1280 PhAN, Nantes).
- Determine stool metagenomic signatures of developmental delay associated with critical CHD.
- Assess acquired brain injury and postnatal exposures relevant to developmental delay. A brain MRI will be performed if clinically indicated in the month after the surgery. It will be performed without anaesthesia, using a standardised protocol and the potential lesions will be described using the validated MRI Abnormality Scoring System.^20^

### Intervention timing and data collection

**Figure 1:**
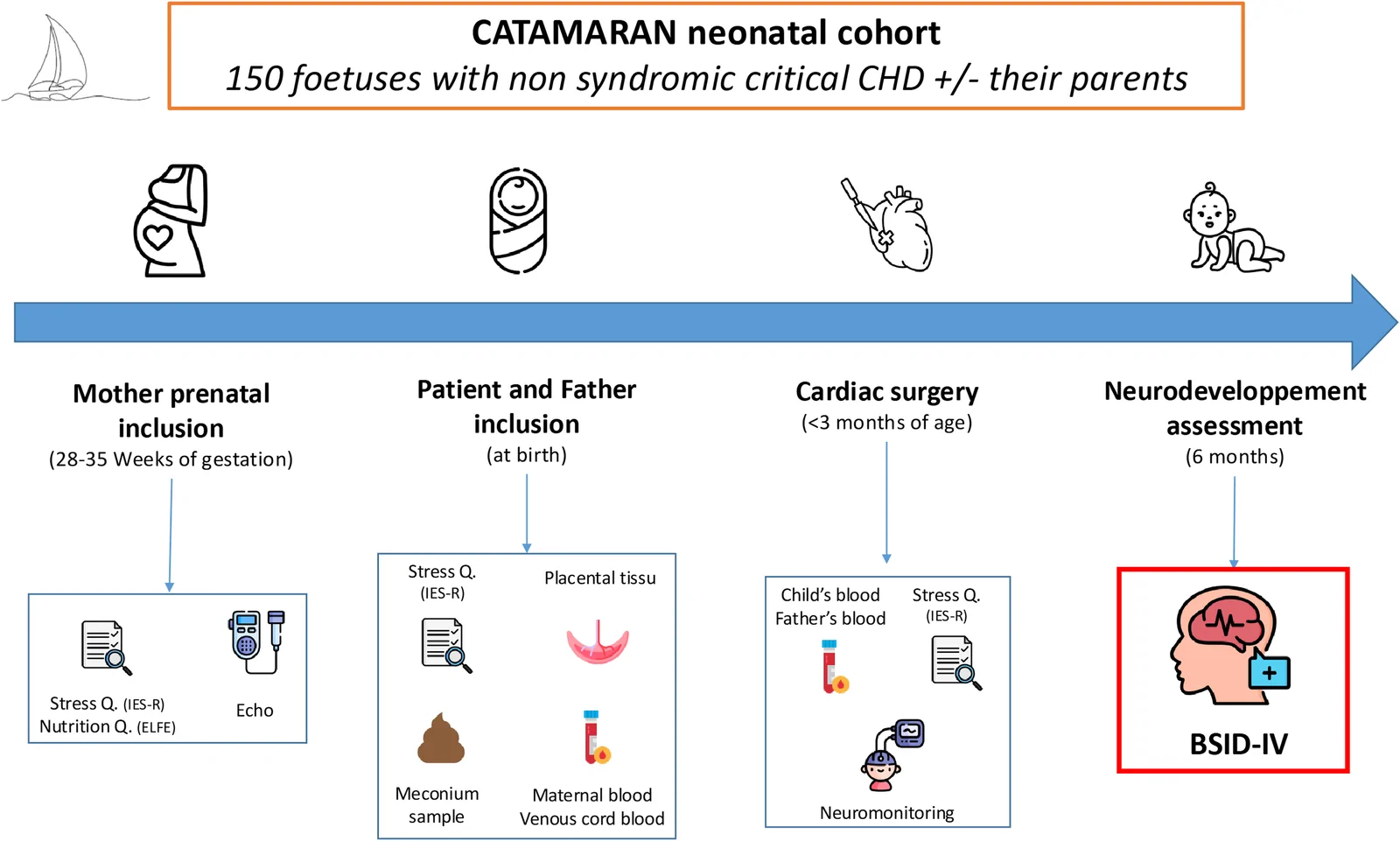
Study intervention. BSID-IV, Bayley Scales of Infant and Toddler Development, Fourth Edition; CHD, congenital heart disease; ELFE, Étude Longitudinale Française depuis l’Enfance; IES-R, impact of event scale-revised; Q, questionnaire.

Family trios will undergo a standardised research protocol with clinical, imaging and biological data to be collected at multiple time points (table 1 and figure 1).

**Table 1:** Data recorded and samples collected

Prenatal period	Current healthcare	Biomedical research
Complete fetal ultrasound (T2)	x	
Fetal functional echocardiography (T2 and T3)	x	
Fetal brain MRI (T3) according to clinical indication		x
Biobanking of blood samples of both parents		x
IES-R questionnaire		x
At birth		
Record of pregnancy history	x	
Postnatal transthoracic echocardiogram	x	
Record of in utero exposures, socio-demographics, ELFE questionnaire		x
IES-R questionnaire		x
Placental gross and histopathological examination		x
Biobanking of maternal blood sample, placenta biopsies, umbilical cord blood sample and newborn’s meconium		x
In-hospital perioperative course		
Record of surgical and ICU course data	x	
12-Lead ECG (baseline and postoperative day 2)	x	
Serial transthoracic echocardiograms	x	
Perioperative neuromonitoring (brain haemodynamics)		x
Postoperative brain MRI according to clinical indication (within 10 days)		x
IES-R questionnaire		x
Biobanking of preoperative and postoperative blood samples (neurobiomarkers)		x
Follow-up		
Serial cardiovascular and neurological clinical examinations	x	
Serial transthoracic echocardiograms	x	
Early neurodevelopmental assessment with Amiel-Tison scale (M3)	x	
Biobanking of stools (M1)		x
IES-R questionnaire (M6)		x
Early neurodevelopmental assessment with BSID-IV (M6)		x

BSID-IV, Bayley Scales of Infant and Toddler Development, Fourth Edition; ELFE, Étude Longitudinale Française depuis l’Enfance; ICU, intensive care unit; IES-R, impact of event scale-revised.

#### Clinical data

Eligible fetuses are identified at the routine mid-trimester morphological ultrasound (18–22 weeks) or a subsequent fetal echocardiogram, with diagnosis confirmed by multidisciplinary team consultation typically at 24–28 weeks of gestation. Maternal inclusion and written informed consent occur at 28–35 weeks of gestation, enabling prenatal sampling close to delivery. Paternal and neonatal inclusion occurs at birth. Follow-up data will be recorded prospectively during pregnancy, at birth, at the surgery, at 3 and 6 months of age. Data on environmental stress exposures (maternal infection, inflammation, smoking, alcohol, substance abuse, medical treatment during the pregnancy) will be collected from the maternal electronic health records. After delivery, anthropometric measurements of mothers and babies, gestational age, prenatal and intrapartum complications (pre-eclampsia, gestational diabetes, fetal growth restriction) and newborn adaptation to extrauterine life parameters will be assessed. The following perioperative variables will be systematically recorded: (i) surgical: CPB duration, aortic cross-clamp time, circulatory arrest duration and temperature, selective cerebral perfusion use, haematocrit and minimum temperature on CPB, transfusion volumes, RACHS-2 score; (ii) ICU/postoperative: duration of mechanical ventilation and inotropic support, lowest mean arterial pressure and peripheral oxygen saturation in the first 48 hours, occurrence of low cardiac output syndrome (need for extracorporeal membrane oxygenation or ≥3 inotropes for >48 hours), acute kidney injury (Kidney Disease: Improving Global Outcomes criteria), seizures, ICU days, total hospital stay; (iii) neurological monitoring: continuous perioperative cerebral near-infrared spectroscopy (regional oxygen saturation, desaturation burden <50%); postoperative brain MRI scored with the MRI Abnormality Scoring System^20^; (iv) neurobiomarkers: plasma neurofilament light chain and glial fibrillar acidic protein on days 0, 1 and 2 postsurgery. Patients will undergo prospective cardiovascular follow-up including serial echocardiograms as per routine practice.

#### Biobanking

Before birth, a mother’s blood sample will be taken. At birth, placenta will be sampled, snap frozen in dry ice and stored at −80°C. At the same time, venous cord blood samples will be collected. Proband’s blood samples will be taken at the time of surgery along with a blood sample from the father for the genetic study (preoperative day 0) and postoperatively (day 1 and day 2) for the postnatal exposure (neurobiomarkers). All these blood samples will be processed within 1 hour and stored at −80°C. Finally, a collection of stools will be realised at birth and at 1 month of age.

All human biological samples (placenta, blood and stools) will be stored in the Biobanking Core Facility at Nantes University Hospital (NF S96-900 and ISO 9001 accreditations), in the existing paediatric biocollection supervised by the study coordinator.

### Statistical analyses

#### Sample size justification

According to the study by Gunn *et al* using the same screening tool in its previous version (Bayley-III), a developmental delay was detected in 32% of patients at the age of 2 years.^21 22^ In our selected population, we expect this rate to be at least as high. To ensure adequate precision of the estimated proportion, we considered a range of possible rates from 5% to 35%. Assuming a 5% loss to follow-up, a total sample size of 150 patients will allow estimation of the proportion of infants with a developmental delay with 95% CIs ranging from 7% to 16% (exact Clopper-Pearson method), depending on the true underlying rate.

#### Statistical analyses

All variables will be described overall and by relevant subgroup. The description will include counts and percentages for qualitative variables and minimum, maximum, mean, SD and median for quantitative variables. The proportion of infants with developmental delay at 6 months, defined as a BSID-IV score ≥1.5 SD below the normative mean in at least one of the measured domains, will be calculated, with 95% CIs estimated using the exact Clopper-Pearson method. To identify factors associated with developmental delay (clinical perinatal, neonatal and postnatal conditions), bivariate analyses will be performed, followed by multivariate logistic regression models adjusting for potential confounders. To explore potential genetic, transcriptomic and other multi-omic factors involved in the occurrence of a developmental delay, a case-control analysis will be conducted within the cohort. Cases will include infants who meet the criteria for developmental delay at 6 months, while controls will be those who do not. Multivariate models will then be applied to account for additional individual risk factors. All tests will be two-sided and the significance threshold will be set at 5%. For multi-omics discovery analyses, a false discovery rate correction (Benjamini-Hochberg method) will be applied independently for each omic stratum. All multi-omics findings will be explicitly reported as hypothesis-generating, pending replication in independent cohorts. Missing data for continuous covariates will be handled by multiple imputation (five imputed datasets, fully conditional specification). A preplanned sensitivity analysis restricted to term-born neonates (≥37 weeks) will assess robustness of the primary endpoint.

#### Potential sources of bias

Selection bias is minimised by consecutive, prospective enrolment with uniform predefined eligibility criteria across all eight centres. Ascertainment bias in the primary outcome is mitigated by blinding the assessing neuropsychologist to diagnosis, neuroimaging and clinical course. Information bias in biological analyses is minimised by standardised biobanking (processing within 1 hour, −80°C storage) and centralised laboratory analyses by operators blinded to outcome data. Participants lost to follow-up will be described and their characteristics compared with completers.

### Patient and public involvement

At what stage in the research process were patients/the public first involved in the research and how? Patients, and particularly the French national association representing parents and patients affected by CHD, Petit Coeur de Beurre, were involved at the screening and inclusion stages. We communicated through the association’s social media platforms to raise awareness of the importance of this research field.

How were the research question(s) and outcome measures developed and informed by their priorities, experience and preferences? Patients and members of the public were not involved in the development of the research question. Therefore, these were not informed by patient or public priorities, experience or preferences.

How were patients/the public involved in the design of this study? Patients and members of the public were not involved in the development of the research question.

How were they involved in the recruitment to and conduct of the study? Patients, and particularly the French national association representing parents and patients affected by CHD, Petit Coeur de Beurre, were involved in the recruitment process at the screening and inclusion stages. We communicated through the association’s social media platforms to raise awareness of the importance of this research field.

Were they asked to assess the burden of the intervention and time required to participate in the research? As the intervention was limited, we decided not to administer a satisfaction questionnaire.

How were (or will) they be involved in your plans to disseminate the study results to participants and relevant wider patient communities (eg, by choosing what information/results to share, when and in what format)? Patients and members of the public will be involved in our plans to disseminate the study results, particularly the clinical study, through social media platforms and meetings of the French national association representing parents and patients affected by CHD, Petit Coeur de Beurre.

### Ethics and dissemination

### Ethics approval

The study will be conducted in compliance with the Good Clinical Practices protocol and Declaration of Helsinki principles. The protocol for the CATAMARAN neonatal study received approval from the ethics committee in France in November 2024 (no. 2024-A00425-42) and the French health authority was informed. Fetuses diagnosed with critical CHD and their two parents will be included in both the CATAMARAN neonatal cohort study and in its biocollection after obtaining informed and written consent from the parents.

### Data collection, storage, monitoring and confidentiality

Data collection for each child and parent participating in the research will be conducted using an electronic case report form (eCRF). Biological samples from all participants will be anonymised with a predefined code before being sent to designated facilities: l’institut du thorax in Nantes (INSERM UMR 1087) for genome and transcriptome sequencing via its genomics core facility (GenoA) and bioinformatics core facility (BiRD) and the PhAN unit (INRAE UMR 1280) for metabolome/lipidome analyses via the mass spectrometry platform (M-Shark), placental epigenetic analysis and metagenome sequencing. All these structures will sign under a collaborative agreement. Each individual responsible for entering data into the eCRF (investigator or research staff) will have a unique user account with specific access rights based on their role, allowing them to enter, modify, lock, monitor or sign eCRF pages. Data will be compiled directly from the eCRF into a secure database hosted on a server with controlled access (account/password) according to user roles. All changes to the data—whether additions, modifications or deletions—will be recorded in a non-editable electronic file (audit trail). Anonymised MRI scan images will be uploaded by investigators and securely stored on a web central database for the duration of the study. All investigators will retain study data for a minimum of 15 years after the study’s conclusion. To maintain confidentiality, each participant will be assigned a code that will be used in the eCRF and all related documents (laboratory samples, MRI scans, etc), serving as the sole means of linking data back to individual participants.

Monitoring of data will follow standard procedures to ensure data quality. If necessary, adjustments and contingency plans will be implemented to address issues such as underinclusion or overinclusion, ensuring that the sample aligns with the predetermined composition and number of participants.

### Dissemination

The *CATAMARAN neonatal* results will be published in high-ranking peer-reviewed journals. Collected data will lead to various publications related to clinical, genetic and placental multi-omics analyses and communications in international scientific meetings.

This project is also supported by the French national association representing parents and patients affected by CHD, namely Petit Coeur de Beurre (https://www.petitcoeurdebeurre.fr/page/43746-l-association). Through our collaboration with this organisation, we have developed thoughtful strategies for engaging with parents participating in the early neurodevelopmental assessment and in the genetic aspect of the study, ensuring our communication is both respectful and sensitive to their experience and personal history. The results of the *CATAMARAN neonatal* project will be disseminated to the general public through this association.
