# Supplementary material for: Clinical and multi-omics characterisation of early neurodevelopmental disorders associated with critical congenital heart disease: the prospective cohort CATAMARAN neonatal study protocol
Source: BMJ Open. 2026 Sep 8;16(9):e116866. doi: 10.1136/bmjopen-2026-116866 (PMC13561013; doi:10.1136/bmjopen-2026-116866)
Supplement: Supplementary file 1 [file bmjopen-2026-116866.reviewer_comments.pdf]

PEER REVIEW HISTORY

BMJ Open publishes all reviews undertaken for accepted manuscripts. Reviewers are asked to complete a checklist review form and are provided with free text boxes to elaborate on their assessment. These free text comments are reproduced below.

ARTICLE DETAILS

Title (Provisional)

Clinical and multi-omics characterization of early neurodevelopmental disorders associated with critical congenital heart disease: the prospective cohort CATAMARAN neonatal study protocol

Authors

Werner, Oscar; Ferchaud-Roucher, Véronique; Karakachoff, Matilde; Bourgoin, Pierre; Chauvire-Drouard, Anne; Galy, Jules; Cosse, Mathilde; Chaffiraud, Mélissa; Boivin, Marion; Gauvard, Evelyne; Egron, Solène; Romefort, Bénédicte; Benbrik, Nadir; Padovani, Paul; Prigent, Solene; Tagorti, Maha; Demonceaux, Marie; Gronier, Céline Grunenwald; Laribi, Jihed; Monier, Astrid; Winer, Norbert; Gaillard-Leroux, Benedicte; Flamant, Cyril; Amarger, Valérie; Moyon, Thomas; Hervé, Blottière; Forest, Virginie; Lindenbaum, Pierre; Dina, Christian; Redon, Richard; Barc, Julien; Ebstein, Frédéric; Schott, Jean-Jacques; Guerra, Amanda; Cadeau, Olivier; Roy, Arnaud; Baruteau, Alban-Elouen; study group, CATAMARAN

VERSION 1 - REVIEW

**Reviewer**	**1**
**Name**	**Saxena, Anita**
**Affiliation**	**All India Institute of Medical Sciences, New Delhi, Cardiology**
**Date**	**30-Apr-2026**
**COI**	**None**

The manuscript defines the study protocol for characterization of neurodevelopmental disorders associated with critical congenital heart diseases (CHD). It is an important research question which is likely to be answered by the study.

I have couple of short comments.

1. Will the study include all fetuses, irrespective of the period gestation at delivery, i.e. preterm babies will also be included?

2. At what stage of gestation will the recruitment be done for the fetus? Generally these anomalies are detected at around 20 weeks of gestation.

3. Neurodevelopmental abnormalities may be related to some of the perioperative factors, can authors elaborate on the various perioperative variables they are planning to include in the analysis.

**Reviewer**	**2**
**Name**	**Hjortdal, Vibeke**
**Affiliation**	**Copenhagen University Hospital Centre for Cardiac Vascular Pulmonary and Infectious Diseases, Department of Cardiothoracic Surgery**
**Date**	**26-May-2026**
**COI**	**None**

Thank you for the opportunity to review this project.

The primary outcome is extensively studied in other publications. 150 patients is a very small cohort.

The aspect of genetic assessment is not realistic with the inclusion of 150 patients.

This study will most likely confirm previous studies in the same field from other countries but it is doubtful that this study will add new confirmatory knowledge.

VERSION 1 - AUTHOR RESPONSE

Reviewer 1

The manuscript defines the study protocol for characterization of neurodevelopmental disorders associated with critical congenital heart diseases (CHD). It is an important research question which is likely to be answered by the study. I have couple of short comments.

1.will the study include all fetuses, irrespective of the period gestation at delivery, i.e. preterm babies will also be included?

We thank the Reviewer for raising this important point of eligibility clarification.

The CATAMARAN study does not restrict inclusion by gestational age at delivery. Both term (≥37 weeks) and preterm neonates are eligible, provided all other inclusion criteria are met. Deliberate exclusion of preterm neonates would substantially compromise the external validity of our cohort, since preterm delivery is a recognized and relatively common complication in fetuses with critical CHD, occurring in approximately 15–20% of cases, reflecting altered fetal hemodynamics and uteroplacental dysfunction (Mebius MJ et al. Pediatr Res. 2017;82(3):423–430; Martínez-Biarge M et al. J Pediatr. 2016;170:137–142).

Gestational age at delivery is treated as a key covariate — not as an exclusion criterion — throughout all analyses. Specifically: (1) gestational age will be recorded prospectively and included in all multivariate regression models; (2) BSID-4 scores will be interpreted using age-corrected norms (corrected age) for all infants born before 37 weeks of gestation, as validated

in preterm CHD populations (Winter EL et al. J Intelligence. 2023;11(11):213); (3) a pre-planned sensitivity analysis restricted to term-born neonates (≥37 weeks) will assess the robustness of the primary findings to the exclusion of preterm infants.

This clarification has been added to Section 3 (Study population) and Section 6 (Statistical analyses) of the revised manuscript.

2.at what stage of gestation will the recruitment be done for the fetus? Generally, these anomalies are detected at around 20 weeks of gestation.

We thank the Reviewer for this pertinent question regarding the recruitment timeline. The Reviewer is correct that critical CHD is typically first suspected at the routine mid-trimester morphological ultrasound scan (18–22 weeks of gestation), with diagnosis usually confirmed by fetal echocardiogram shortly thereafter.

In the CATAMARAN protocol, the prenatal recruitment sequence is as follows: (i) Fetal diagnosis is confirmed by the multidisciplinary team (fetal cardiologist, pediatric cardiologist, cardiac surgeon) during the team consultation typically held between 24 and 28 weeks of gestation. (ii) Maternal inclusion and consent occur between 28 and 35 weeks of gestation, which corresponds to the time window for prenatal maternal blood sampling (biobanking) and administration of the IES-R stress questionnaire and ELFE nutrition questionnaire. This window was chosen to ensure the maternal biological samples are collected sufficiently close to delivery to capture the third-trimester fetoplacental metabolic environment, whilst allowing adequate time for consent procedures. (iii) Fetal/neonatal and paternal inclusion occurs at birth.

We acknowledge that the diagnosis itself is typically established earlier (18–28 weeks), and that the 28–35 week inclusion window represents a pragmatic balance between biological timing and consent feasibility. This has now been clarified explicitly in Section 3 of the revised manuscript.

3.Neurodevelopmental abnormalities may be related to some of the perioperative factors, can authors elaborate on the various perioperative variables they are planning to include in the analysis

We thank the Reviewer for this important question. The perioperative period is indeed a critical exposure window for brain injury and NDD in critical CHD infants. The CATAMARAN study captures an extensive set of perioperative variables, which are now detailed explicitly in Section 5 (Data collection) of the revised manuscript.

Surgical variables: type of cardiac surgery (palliative vs. corrective), cardiopulmonary bypass (CPB) duration (minutes), aortic cross-clamp time (minutes), circulatory arrest duration and temperature, use of selective cerebral perfusion, hematocrit on CPB, minimum temperature on CPB, transfusion requirements (packed red blood cells, fresh frozen plasma, platelets), and RACHS-2 surgical risk score.

ICU/postoperative clinical variables: duration of mechanical ventilation, duration of inotropic support, lowest mean arterial pressure in the first 48 hours, lowest arterial oxygen saturation (SpO_2) in the first 48 hours, occurrence of low cardiac output syndrome (defined as need for

ECMO or ≥3 inotropes for >48 hours), occurrence of acute kidney injury (KDIGO criteria), occurrence of seizures, number of ICU days, total hospital length of stay.

Neurological monitoring and imaging: continuous perioperative near-infrared spectroscopy (NIRS) for cerebral regional oxygen saturation (rSO_2) monitoring, documenting desaturation burden (area under the curve below threshold of 50%); postoperative brain MRI (if performed for clinical indication within 10 days of surgery), scored using the validated MRI Abnormality Scoring System (MASC) (Stegeman R et al. Am J Neuroradiol. 2021;42(11):2034–2039).

Neurobiomarkers: pre- and postoperative blood samples (day 0, day 1, day 2) for candidate neurobiomarkers of brain injury, including neurofilament light chain (NfL) and glial fibrillary acidic protein (GFAP), in line with recent evidence from the CHD field (Ferriero DM et al. Lancet Neurol. 2020;19(4):341–352).

All perioperative variables will be entered in the case report form (eCRF) by the local co-investigator and will be included as covariates in multivariate models of neurodevelopmental outcome. This complete list has been added to Section 5 of the revised manuscript.

Reviewer 2

Thank you for the opportunity to review this project. The primary outcome is extensively studied in other publications.

We respectfully disagree with the Reviewer's assessment, and thank them for the opportunity to clarify the multidimensional scientific contribution of CATAMARAN beyond its primary descriptive objective.

The Reviewer is correct that the prevalence of neurodevelopmental delay (NDD) in critical CHD has been estimated in prior cohort studies — principally using Bayley-III in Anglo-Saxon or Australian centers (Gunn JK et al. Arch Dis Child. 2016;101:1010–1016; Long SH et al. Early Hum Dev. 2012;88:603–607). However, three points distinguish the CATAMARAN primary endpoint from a mere replication exercise:

First, France has no existing multicenter, prospective cohort study measuring the prevalence of NDD in critical CHD infants using a standardized assessment tool. The current evidence base for France comes exclusively from single-center retrospective series. A French multicentric estimate is needed to guide national healthcare policy and support the development of a coordinated, evidence-based early intervention program — a recognized gap documented by our group (Cadeau O et al. Pediatr Res. 2025; doi:10.1038/s41390-025-04390-6).

Second, the use of BSID-4 (the fourth edition) is itself novel: virtually all existing CHD cohort data were obtained with BSID-III, whose normative ceiling is known to overestimate developmental scores. The BSID-4 recalibration produces systematically lower scores (approximately 7–10 points lower in the cognitive domain), such that CATAMARAN will provide the first French estimate based on contemporary normative standards.

Third — and most importantly — the primary descriptive objective is explicitly positioned as the methodological foundation for the deeply innovative secondary multi-omics objectives described below. The primary outcome is what defines the case-control stratification for all molecular analyses.

150 patients is a very small cohort. The aspect of genetic assessment is not realistic with the inclusion of 150 patients. This study will most likely confirm previous studies in the same field from other countries but it is doubtful that this study will add new confirmatory knowledge.

We thank the Reviewer for this critique, which gives us the opportunity to articulate the design rationale and the specific scientific ambitions of the CATAMARAN multi-omics strategy.

— ON SAMPLE SIZE FOR THE PRIMARY OBJECTIVE —

The sample size of 150 was calculated for the primary endpoint: estimating the proportion of developmental delay using the exact Clopper-Pearson method with pre-specified precision. This is a descriptive estimate, not a comparative hypothesis test, and 150 subjects provides 95% confidence intervals of acceptable width (±7–9%) across a range of plausible prevalence estimates (5–35%). This is consistent with the statistical approach used in analogous prevalence cohorts in rare pediatric diseases.

— ON SAMPLE SIZE FOR MULTI-OMICS EXPLORATORIES —

The multi-omics analyses are explicitly designed as discovery/hypothesis-generating investigations, not as confirmatory analyses. In this context, the sample size of 150 (yielding an estimated 42–52 cases assuming a 28–35% NDD rate) is well within the range used in comparable published multi-omics discovery studies in CHD and related fields. For comparison: the landmark placental transcriptomics study in CHD by Westermeier et al. (Sci Rep. 2021;11:16436) comprised 30 samples; the epigenomic profiling study by Bos et al. (Epigenetics. 2022;17(9):965–982) used 20–40 subjects; metabolomic studies in CHD placentas (Maslen CL et al. Pediatr Res. 2019) involved <50 subjects. CATAMARAN therefore represents one of the largest prospective multi-omics CHD datasets assembled to date. False discovery rate correction (Benjamini-Hochberg) will be applied independently for each omic layer, and all findings will be explicitly reported as hypothesis-generating pending external replication.

— ON THE UNIQUE INNOVATION OF THE PLACENTAL MULTI-OMICS CASE-CONTROL APPROACH —

The CATAMARAN placental multi-omics strategy is, to our knowledge, unprecedented in the field of CHD-associated NDD. No published study has simultaneously applied multiple omic layers to human placental tissue in the specific context of CHD-associated NDD, using neurodevelopmental outcome as the case-control stratifying variable. The layers integrated in CATAMARAN are: (1) transcriptomics (RNA-sequencing) to identify differentially expressed genes and dysregulated pathways — notably mitochondrial bioenergetics, inflammatory signaling, and angiogenesis — in cases versus controls; (2) epigenomics (DNA methylation profiling) on placental biopsies and umbilical cord blood mononuclear cells, targeting differentially methylated regions at imprinted and developmentally regulated loci that may encode in utero neurodevelopmental programming; (3) metabolomics and lipidomics (detailed below); (4) metagenomics on meconium (gut-brain axis). The placenta is uniquely positioned at the interface between the maternal environment, the fetal cardiovascular system, and brain development, and its systematic molecular characterization in this clinical context opens a

genuinely new pathophysiological paradigm: the cardiac-placental-brain triad within the DOHaD framework (Demonceaux M et al. JACC Basic Transl Sci. 2025;doi:10.1016/j.jacbts.2025.01.022).

— ON THE UNIQUE INNOVATION OF THE MATERNAL-PLACENTAL-FETAL LIPIDOMIC TRIAD —

The simultaneous, integrated lipidomic profiling of three biological compartments — (i) maternal peripheral blood (28–35 weeks), (ii) placental tissue (at delivery), and (iii) umbilical cord blood / early neonatal blood — constitutes an entirely novel design in the CHD-NDD field. To our knowledge, no published study has characterized this full maternal-placental-fetal lipidomic continuum in critical CHD with neurodevelopmental outcome as the stratifying case-control variable. The scientific rationale is mechanistically robust: lipids — in particular long-chain polyunsaturated fatty acids (LC-PUFAs), lysophospholipids, and sphingolipids — are not merely energy substrates but critical signaling mediators of trophoblast function, fetoplacental nutrient transfer, myelination, and synaptic plasticity (Innis SM. J Nutr. 2007;137(4):855–859; Bazinet RP & Layé S. Nat Rev Neurosci. 2014;15(12):771–785). By integrating the maternal nutrition questionnaire (FFQ-ELFE) — collected at the same time point as maternal blood sampling — CATAMARAN will further model the dietary contribution to the lipidomic triad, an analytical dimension absent from all prior studies. This triadic design is entirely original and has direct translational relevance: identification of specific lipidomic signatures could ultimately inform nutritional interventions during pregnancy.
